# Polypharmacy among HIV positive older adults on anti-retroviral therapy attending an urban clinic in Uganda

**DOI:** 10.1186/s12877-018-0817-0

**Published:** 2018-05-29

**Authors:** Michael Ssonko, Fiona Stanaway, Harriet K. Mayanja, Tabitha Namuleme, Robert Cumming, John L. Kyalimpa, Yvonne Karamagi, Barbara Mukasa, Vasi Naganathan

**Affiliations:** 1 Mildmay Uganda, 12 Km Entebbe Road, Naziba Hill Lweza, P.O. Box 24985, Kampala, Uganda; 2 Makerere University College of Health Sciences, Kampala, Uganda; 3 Sydney School of Public Health, Sydney Medical School, University of Sydney, Sydney, Australia; 4 Centre for Education and Research on Ageing, Sydney Medical School, Concord Clinical School, University of Sydney, Sydney, Australia; 5 Ageing and Alzheimers Institute, Concord Hospital, Sydney, Australia

**Keywords:** Polypharmacy, HIV, Drug-related side effects and adverse reactions

## Abstract

**Background:**

Polypharmacy has not been investigated in patients living with HIV in developing countries. The aims of this study were to determine the prevalence of polypharmacy, the factors associated with polypharmacy and whether polypharmacy was associated with adverse effects among older adults on anti-retroviral therapy (ART).

**Methods:**

Cross-sectional study in older adults aged 50 and over on ART attending an outpatient HIV/AIDS care centre in Uganda. Demographic and clinical data collected on number and type of medications plus supplements, possible medication related side-effects, comorbidity, frailty, cognitive impairment, current CD4 count and viral load.

**Results:**

Of 411 participants, 63 (15.3, 95% C.I. 11.9, 18.8) had polypharmacy (≥ 4 non- HIV medications). In multivariate analyses, polypharmacy was associated with one or more hospitalisations in the last year (Prevalence Ratio PR = 1.8, 95% C.I. 1.1, 3.1, *p* = 0.02), prescription by an internist (PR = 3.6, 95% C.I. 1.3, 10.5, p = 0.02) and frailty index scores of 5 to 6 (PR = 10.6, 95% C.I. 1.4, 78, p = 0.02), and 7 or more (PR = 17.4, 95% C.I. 2.4, 126.5, *p* = 0.005). Polypharmacy was not associated with frequency and severity of possible medication related side effects and falls.

**Conclusion:**

Polypharmacy is common among older HIV infected patients in sub-Saharan Africa. It’s more prevalent among frail people, who have been in hospital in the last year and who have been seen by an internist. We found no evidence that polypharmacy results in any harm but this is worth exploring further.

**Electronic supplementary material:**

The online version of this article (10.1186/s12877-018-0817-0) contains supplementary material, which is available to authorized users.

## Background

Polypharmacy is common among HIV positive older adults on anti-retroviral therapy (ART) compared to younger people with HIV in western countries [1–3]. In a Swiss cohort study, 14% of HIV-positive participants older than 65 years were taking 4 or more non-HIV medications [3]. Some of the common classes of concomitant drugs taken by older people with HIV are antihypertensive medicines, analgesics [4, 5] as well as supplements and herbal medications [6].

HIV-infected older adults are potentially at increased risk of medication-related adverse effects from non-HIV medications [7, 8]. Nachega et al. suggests that the combination of ART and polypharmacy increases the chances of serious drug-drug interactions, poor ART adherence, loss of efficacy of the co-administered medication, or virologic breakthrough [9].

Studies looking at ART and polypharmacy however, have been carried out in western countries that have more access to medications compared to sub-Saharan Africa. This is the region with the highest burden of HIV [10, 11] where the prevalence of HIV infection among the 74 million people aged ≥50 years is 4.0% [12]. In these countries we do not know if polypharmacy is common among older people on HIV treatment and if polypharmacy results in any harm. The aim of this study therefore was to determine the prevalence of polypharmacy, the factors associated with polypharmacy and whether polypharmacy was associated with adverse effects among older adults on ART at an urban HIV clinic in Uganda.

## Methods

Cross-sectional data was collected between March and July 2015 from ambulatory older adults aged 50 and over on ART attending Mildmay Uganda outpatient HIV/AIDS care centre (11.5 Km south of the capital, Kampala). Mildmay offers care to over 12,500 clients of which 1206 (9.6%) are aged 50 and over. HIV related services are provided at no cost to patients. Systematic random sampling was used for enrollment into the study. Clients on ART to be seen for the day were randomly arranged in a database. The sampling interval (n) was derived through dividing the total number of persons aged 50 years and over on ART scheduled for the day (e.g. 30) by the number to be enrolled in a given day (e.g. 10). Counting down the randomly generated list from the first subject, every nth (e.g. 3rd) client was invited to take part in the study on the day of their routine clinic appointment prior to the scheduled consultation with the clinician. They were then informed about the study, its purpose, benefits, risks/hazards and what was required from them. Written consent from client was later obtained. Participants unable to attend the interview session were excluded from the study.

### Ethical approval

The study was approved by the Mildmay Uganda Research and Ethics Committee. Permission to do the study was obtained from Uganda National Council for Science and Technology.

### Demographic and medical details

Data was collected by interview in a confidential setting using a pre-coded, pre-tested standardized questionnaire. Demographic data included gender, age, source of income, owned or rented residential premises, level of education, marital status, living arrangements (living alone or with others) and distance lived from the clinic. Charlson’s weighted index of comorbidity was used to quantify comorbidity [13]. Frailty was assessed using a 40 -item frailty index questionnaire which was practical for use in our clinic [14]. Cognition impairment and performance status were assessed using Folstein’s Mini Mental State Examination and the Karnofsky rating criteria questionnaires respectively. Participants were asked about the number of hospitalisations in the previous year and number of falls they had in the previous 12 months [15].

### Medications and possible medication related adverse events

Participants were asked about conventional medications, complementary medications (“general well-being” and multi-use preparations which are usually herbs), vitamins and nutritional supplements they were taking. Verification of this information was done by checking the previous clinic visit prescriptions as documented in the case file. This included the type and duration of current ART. Any other prescriptions were scrutinised along with any medications or supplements that the participants had brought with them.

Polypharmacy was defined as the use of four or more non – HIV medications, complimentary medications or supplements within the period from the previous visit. Clinic visit intervals are between one to 3 months. HIV and HIV- related medication including ART and cotrimoxazole were excluded in determining polypharmacy.

Prescribing cadre refers to who prescribed at the time of the last clinic visit. These included nurses, physician assistants, general medical officers and internists (physician in internal medicine).

Assessment of adherence to ART, using the ministry of health criteria [16] was based on self-report and pill counts. Frequency and severity of possible medication related side-effects was based on an un-validated questionnaire that we derived (Additional file 1: Figure S1). Within the time frame of clinic visits it was not possible to administer a more detailed medication adverse effects questionnaire.

### Blood results

Results of the most recent CD4 count within the prior 6 months of interview date and most recent viral load within the prior 1 year from interview date were collected to investigate if polypharmacy had an effect on immunological response and viral suppression respectively.

### Data analysis

Analysis was conducted in SAS version 9.4 (SAS Institute, Inc., Cary, NC). Characteristics of participants by the presence or absence of polypharmacy were compared using the chi-squared test for categorical variables and the t test for continuous variables that demonstrated a linear relationship with polypharmacy. As polypharmacy is a common outcome (frequency > 10%) an odds ratio is not an appropriate measure of effect and so poisson regression was used to estimate unadjusted and adjusted prevalence ratios for characteristics associated with polypharmacy [17]. In the main analysis, the dependent variable was polypharmacy and independent variables included socio-demographic characteristics and clinical features with a reported association with polypharmacy. All variables with *P* < 0.2 in univariate analyses were included in the baseline multivariate model. Non-significant variables (*p* > 0.05) were removed from the model by backward stepwise elimination to obtain the final model.

Additional analyses using Poisson regression and backward stepwise elimination of non-significant variables were conducted to determine the presence of independent associations between polypharmacy and potential negative effects of polypharmacy. The dependent variables in these analyses were ART adherence, viral load, frequency of medication related side effects, severe medication related side effects, and falls. Independent variables included polypharmacy and other socio-demographic and clinical characteristics known to be associated with the outcome of interest.

The sample size was estimated on the prevalence outcome using the Leslie Kish formula [18]. The prevalence of polypharmacy was estimated as 40% [19] and an assumption of 10% ineligible participant data was made to cater for eligibility violations and other uncertainties. On these assumptions a sample size of 400 was chosen with the aim of being able to estimate the prevalence with a 95% confidence interval (precision) of ±5%.

## Results

**Fig. 1:**
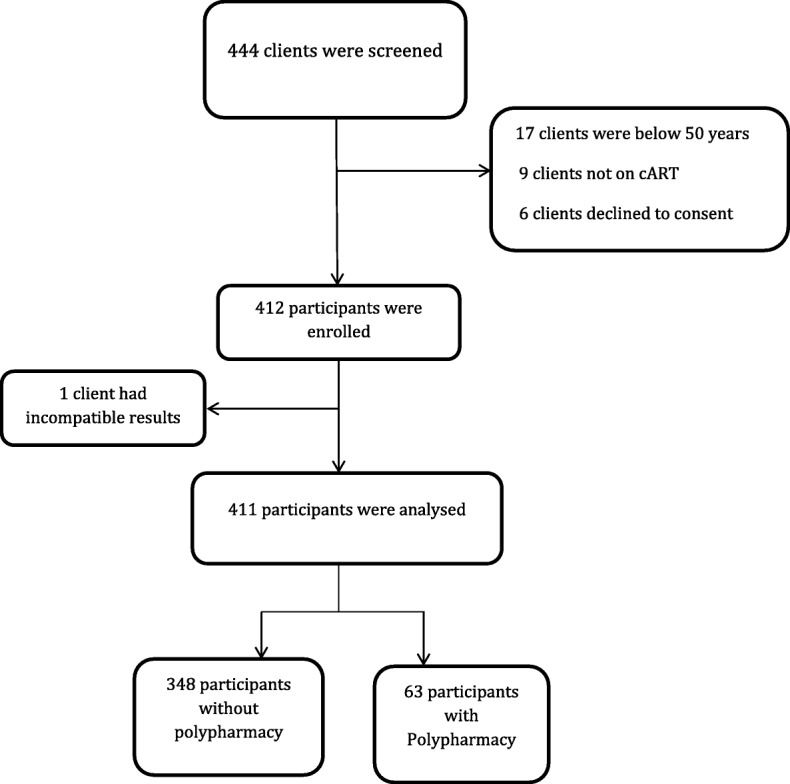
Screening and recruitment of HIV positive older adults on ART into the study

Between March 2015 and July 2015, 444 patients were approached to participate in the study of whom 33 (7.4%) were not included: six declined, 17 were below age of 50 years, nine were not on ART, and one had missing or incompatible results as shown in Fig. 1. The total number of participants analysed was 411 of which 63 (15.3, 95% C.I. 11.9, 18.8) had polypharmacy. The most commonly used medicines among all participants and the subgroup with polypharmacy were antihypertensive agents, diuretics and non-steroidal anti-inflammatory medications as shown in Table 1.

**Table 1:** Most commonly used medicine classes in all study participants and those with polypharmacy

All Participants		Participants with Polypharmacy	
Most commonly used medicine classes (*n* = 636 total different medicines used among 411 participants)		Most commonly used medicine classes (*n* = 289 total different medicines used among 63 participants with polypharmacy)	
Drug Class	n (%)	Drug Class	n (%)
Antihypertensive agents	133 (20.9)	Antihypertensive agents	72 (24.9)
Diuretics	92 (14.5)	Diuretics	48 (16.6)
Non-steroidal anti-inflammatory agents	51 (8.0)	Non-steroidal anti-inflammatory agents	17 (5.9)
B group vitamins and minerals.	35 (5.5)	B group vitamins and minerals	16 (5.5)
General well-being, multiple use preparations like herbs, others	31 (4.9)	Beta-adrenergic blocking agents	13 (4.5)
Antidepressants	30 (4.7)	Hyperacidity, reflux and ulcers	12 (4.2)
Hyperacidity, reflux and ulcers	28 (4.4)	General well-being, multiple use preparations like herbs, others	10 (3.5)
Beta-adrenergic blocking agents	27 (4.3)	Antidepressants	9 (3.1)
Simple analgesics and antipyretics	24 (3.8)	Hypoglycaemic agents	9 (3.1)
Hypoglycaemic agents	16 (2.5)	Simple analgesics and antipyretics	8 (2.8)

n = Absolute numbers; % = percentage

In univariate analysis (see Table 2), female participants were more likely to have polypharmacy (19% vs. 10%; PR =1.9, 95% C.I. 1.2, 3.3) compared to males. Participants aged 65 years and above were more likely to have polypharmacy (PR =2.6, 95% C.I. 1.5, 4.5) than those aged 50 to 54 years. Participants who had one or more hospitalisations in the previous year (PR = 2.9, 95% C.I. 1.7, 4.8), had an internist’s prescription (PR = 7.3, 95% C.I. 2.6, 20.8), higher comorbidity and higher frailty index scores were also more likely to have polypharmacy. Polypharmacy was less likely in participants who lived in rented residences (PR = 0.4 (95% C.I. 0.1, 0.8). In multivariate analyses (Table 3), the only characteristics associated with polypharmacy were one or more hospitalisations in the last year (PR = 1.8, 95% C.I. 1.1, 3.1, *p* = 0.02), prescription by an internist (PR = 3.6, 95% C.I. 1.3, 10.5, *p* = 0.02) and frailty scores of 5 to 6 (PR = 10.6, 95% C.I. 1.4, 78, *p* = 0.02), and 7 or more (PR = 17.4, 95% C.I. 2.4, 126.5, *p* = 0.005). Polypharmacy was associated with CD4 count of ≥350 cells/μl (PR = 2.38, 95% C.I. 1.17, 4.86, *p* = 0.02) in univariate analysis but not in multivariate analyses (results not shown in tables).

**Table 2:** Characteristics of study participants by polypharmacy (univariate analyses)

Characteristic	Polypharmacy n (%)	No Polypharmacy n (%)	Unadjusted prevalence ratio (95% CI)
Sex			
Male	17 (10)	155 (90)	1
Female	46 (19)	193 (81)	1.9 (1.2–3.3)
Age (years)			
50–54	22 (13)	148 (87)	1
55–59	12 (9)	116 (91)	0.7 (0.4–1.4)
60–64	11 (18)	49 (82)	1.4 (0.7–2.7)
≥ 65	18 (34)	35 (66)	2.6 (1.5–4.5)
Residence			
Own	50 (18)	223 (82)	1
Rented	5 (6)	73 (94)	0.4 (0.1–0.8)
Other	8 (13)	52 (87)	0.7 (0.4–1.5)
Approximate distance from clinic (km)			
0–4.9	5 (12%)	36 (88%)	1
5–9.9	13 (22%)	46 (78%)	1.8 (0.7–4.7)
10–14.9	7 (9%)	72 (91%)	0.7 (0.3–2.2)
≥ 15	38 (16%)	194 (84%)	1.3 (0.6–3.2)
Marital status			
Married	20 (13%)	129 (87%)	1
Widowed	29 (19%)	123 (81%)	1.5 (0.9–2.5)
Divorced	14 (14%)	87 (86%)	1.1 (0.6–2.1)
Single	0 (0%)	8 (100%)	Not applicable
Living arrangements			
Lives alone	8 (15)	46 (85)	1
Lives with others	55 (15)	302 (85)	1.0 (0.5–1.9)
Education level			
Illiterate	5 (16)	26 (84)	1
Primary	30 (16)	163 (84)	1.0 (0.4–2.3)
Secondary	16 (14)	99 (86)	0.9 (0.3–2.2)
Tertiary	12 (17)	60 (83)	1.0 (0.4–2.7)
Source of income			
Active	48 (15)	280 (85)	1
Passive	15 (18)	68 (82)	0.8 (0.5–1.4)
Hospitalisations in previous year			
None	51 (13)	329 (87)	1
One or more	12 (39)	19 (61)	2.9 (1.7–4.8)
Prescribing cadre			
Nurse	4 (9)	40 (91)	1
Physician assistant	25 (12)	177 (88)	1.4 (0.5–3.7)
General medical officer	28 (19)	123 (81)	2.0 (0.8–5.5)
Internist	6 (67)	3 (33)	7.3 (2.6–20.8)
Duration since Highly Active Anti – Retroviral Therapy commenced			
< 5 years	18 (12%)	127 (88%)	1
≥ 5 years	36 (15%)	201 (85%)	1.2 (0.7–2.1)
Charlson’s weighted comorbidity score			
0	29 (10%)	253 (90%)	1
1	23 (22%)	82 (78%)	2.1 (1.3–3.5)
≥ 2	11 (46%)	13 (54%)	4.5 (2.6–7.8)
Frailty index score			
0–2	1 (2)	62 (98)	1
3–4	8 (6)	132 (94)	3.6 (0.5–28.2)
5–6	22 (19)	95 (81)	11.8 (1.6–85.8)
≥ 7	32 (35)	59 (65)	22.2 (3.1–157.9)

*CI* Confidence Interval, *Km* Kilometres

**Table 3:** Characteristics associated with polypharmacy in multivariate adjusted analysis

Characteristic	Adjusted PR (95% CI)	*P* value
Hospitalisation		
None	1	
One or more hospitalisations in last year	1.8 (1.1–3.1)	0.02
Prescribing cadre		
Nurse	1	
Clinical officer	1.0 (0.4–2.6)	0.99
Medical officer	1.4 (0.5–3.7)	0.49
Internist	3.6 (1.3–10.5)	0.02
Frailty score		
0–2	1	
3–4	3.3 (0.4–25.6)	0.25
5–6	10.6 (1.4–78.0)	0.02
7 or more	17.4 (2.4–126.5)	0.005

*PR* Prevalence Ratio, *CI* Confidence Interval

Table 4 shows the factors associated with three outcomes that are possible adverse effects of polypharmacy: frequency and severity of possible medication side effects and falls. In univariate analyses, polypharmacy was associated with one or more possible medication related side effects once or more per week and with moderate or severe distress due to one or more possible medication related side effects but these associations were no longer significant in the multivariate analyses. Polypharmacy was not associated with falls. In addition, polypharmacy was not associated with adherence to ART or unsuppressed viral loads in univariate analyses (data not shown). Frailty was significantly associated with both medication side effects outcomes and falls in multivariate analyses. Mild and moderate cognitive decline was associated with falls. In multivariate analyses being on a protease inhibitor regimen was associated with frequency of possible medication related side effects and falls.

**Table 4:** Predictors of Possible Adverse effects of Medications

Characteristic	Unadjusted PR (95% CI)	*P* value	Adjusted PR (95% CI)	*P* value
≥1 possible side effect due to medication once or more per week				
Polypharmacy	1.7 (1.2–2.3)	0.002	1.2 (0.8–1.6)	0.38
Protease inhibitor based regimen	1.7 (1.1–2.4)	0.01	1.6 (1.1–2.3)	0.01
Frailty score				
0–2	1		1	
3–4	2.1 (1.0–4.5)	0.05	2.1 (1.0–4.5)	0.05
5–6	2.9 (1.4–6.2)	0.005	2.9 (1.4–6.1)	0.006
7 or more	4.7 (2.3–9.8)	< 0.001	4.4 (2.1–9.4)	< 0.001
Moderate or severe distress of ≥1 medication side effect				
Polypharmacy	1.9 (1.3–2.8)	0.002	1.1 (0.7–1.7)	0.57
Frailty score				
0–2	1			
3–4	1.4 (0.5–3.6)	0.54	1.3 (0.5–3.5)	0.55
5–6	2.8 (1.1–6.9)	0.03	2.7 (1.1–6.8)	0.03
7 or more	5.7 (2.4–13.6)	< 0.001	5.4 (2.2–13.4)	< 0.001
≥1 Fall in previous 12 months				
Polypharmacy	1.6 (0.9–2.9)	0.09	1.0 (0.6–1.6)	0.90
Protease inhibitor based regimen	1.9 (1.0–3.6)	0.06	1.9 (1.1–3.3)	0.03
Frailty score				
0–2	1		1	
3–4	2.3 (0.5–10.0)	0.29	2.3 (0.5–10.4)	0.28
5–6	5.1 (1.2–21.3)	0.02	5.2 (1.2–22.3)	0.02
7 or more	9.0 (2.2–36.6)	0.002	7.8 (1.9–32.4)	0.005
Cognitive status (using MMSE^a^)				
Normal cognition	1		1	
Mild cognitive decline	2.4 (1.2–4.6)	0.009	2.0 (1.1–3.9)	0.03
Moderate cognitive decline	4.1 (2.4–6.8)	< 0.001	3.6 (2.2–5.9)	< 0.001
Severe cognitive decline	1.4 (0.5–4.4)	0.53	1.1 (0.4–3.4)	0.87

^a^*Mini* Mental State Examination, *PR* Prevalence Ratio

## Discussion

We found a moderate prevalence of polypharmacy of non-HIV medicines (15%) among HIV positive older adults on ART in this cross-sectional study. This is comparable to the findings of a Swiss cohort study which found a prevalence of 14% of polypharmacy (≥ 4 medications) in those aged > 65 [3]. It is unclear however if the non-HIV medication count in the Swiss study included supplements, herbal preparations and vitamins [3]. The prevalence of polypharmacy in our study and the Swiss study is much lower than in a study of community dwelling predominantly male HIV infected adults aged ≥ 60 in the San Francisco area which found a prevalence of polypharmacy (≥ 5 medications) of 74% [8]. In this case, vitamins, minerals, herbal medications and other supplements were included. We are not aware of any published studies that have looked at non-HIV medications in an older population living with HIV from a developing country. We think it is interesting that antihypertensive medications were the most common non-HIV class of medications taken by our study participants which is similar to what has been found in cohorts from developed countries [2, 5].

Our study showed that frailty, hospitalisation in the last year and being seen in the clinic by an internist were associated with polypharmacy. Internists at the outpatient clinic usually review more complex patients that may be frail with multi-comorbidity. The number of comorbidities and age ≥ 65 were associated with polypharmacy in univariate analyses but were not retained in the multivariate models most likely because the most parsimonious multivariate model would not include both frailty index score and comorbidity score. Polypharmacy was associated with a frailty index score of 5 and above. This relatively low score could have been due to the fact that in the busy HIV clinic the diagnoses for some of the 40 frailty index parameters like dementia, glaucoma, and chronic obstructive pulmonary disease may not have been made, even if present. Living in rented accommodation was associated with being less likely to have polypharmacy. Although not significantly associated with polypharmacy in the multivariate analyses, it does raise the question about whether ability to afford medications, supplements and vitamins influences the number of medications taken. We did not ask directly about income.

The harms of polypharmacy in older people are well established to the point where current research in this area is focused on determining the benefits of deprescribing [20]. In older HIV infected clients however there have only been a few studies such as ours that have looked at whether polypharmacy is harmful in terms of clinical outcomes. A retrospective study in New Orleans, found that the number of medications, including ART, was associated with falls [21]. In our study we did not find an association between polypharmacy and possible adverse effects of medications such as falls or adverse symptoms that could be due to medications. On the other hand, higher frailty scores were associated with possible medication related symptoms and falls.

There has been a view that polypharmacy could result in decreased compliance with ART [22]. We did not find this. An interesting alternative view is that older HIV patients have better adherence to ART than younger people that in turn might increase the risk of adverse effects due to potential drug-drug interactions between ART and other medications [8, 23, 24].

It is possible that the lack of negative effects of polypharmacy on adherence and side effects in this population could be due to the setting. Most previous research on polypharmacy has been done in western countries where people are more likely to take lots of medications with greater potential for harm. It is possible that in a setting where access to medicines is reduced, the decision making process around prescribing is different with a resulting increase in the benefit to harm ratio. The finding that living in rented accommodation was negatively associated with polypharmacy in univariate analyses supports the notion that income may influence the number of non-HIV medications taken.

We found severity of medication side effects and frequency of side effects to be more likely in frail participants, consistent with other studies [25]. In keeping with our finding, a study in the United states found an association between impaired cognition and falls as well as frailty and falls in people with HIV [26]. The association between protease inhibitors and medication adverse effects could be explained by the complex cytochrome P450 inhibition and induction effects that occur with protease inhibitors as well as effects on P-glycoprotein and other drug transporters that increase risk for interactions with HIV or non-HIV related drugs [27].

Our study is the first to describe the prevalence of polypharmacy and factors associated with it in an older representative sample of patients seen in an HIV clinic in sub–Saharan Africa. Our study however is a cross-sectional study and our sample size is relatively small and so we cannot rule out the fact that a larger study may have found differences in the factors significantly associated with polypharmacy. A prospective study in this population would be the better design to determine if polypharmacy is associated with adverse effects. A major limitation of our study is that we used an un-validated tool to determine medication side effects where the symptoms asked about are not specific to medication related adverse effects and could be disease related. In addition, future studies in this population and setting should try to make the distinction between appropriate and inappropriate polypharmacy [28].

## Conclusion

Our study suggests polypharmacy is common among older HIV infected patients in sub-Saharan Africa. Polypharmacy was more prevalent among frail people, who have been in hospital in the last year and who are seen by an internist in the HIV clinic. As older people with HIV in sub-Saharan Africa live longer and become more affluent, it is likely that polypharmacy will become more prevalent. Keeping the limitation of the study design in mind, we did not find evidence that polypharmacy results in any harm but this is worth exploring further.

## Additional file

Additional file 1: Medication side-effects questionnaire used in study where the participant was asked to indicate any symptoms s/he has which s/he believes is a side-effect of the medication s/he is taking. Medication side-effects questionnaire. (DOCX 18 kb)

## Abbreviation

ART: Anti-Retroviral Therapy
