# Supplementary material for: Polypharmacy among HIV positive older adults on anti-retroviral therapy attending an urban clinic in Uganda
Source: BMC Geriatr. 2018 May 29;18:125. doi: 10.1186/s12877-018-0817-0 (PMC5975487; doi:10.1186/s12877-018-0817-0)
Supplement: Supplementary file 1 — Medication side-effects questionnaire used in study where the participant was asked to indicate any symptoms s/he has which s/he believes is a side-effect of the medication s/he is taking. Medication side-effects questionnaire. (DOCX 18 kb) [file 12877_2018_817_MOESM1_ESM.docx]

# Medication side-effects questionnaire used in study where the participant was asked to indicate any symptoms s/he has which s/he believes is a side-effect of the medication s/he is taking

| **SYMPTOMS** | **FREQUENCY** | **SEVERITY** | **COMMENTS** |
| --- | --- | --- | --- |
|  | 0 - not at all  1 - not during past month  2 - less than once per week  3 - once or more per week | 0 (none) - not experienced/ no distress  1 (mild) - causes minor distress only  2 (moderate) - moderately distressing  3 (severe) - causes significant distress |  |
| Nausea | 0 1 2 3 | 0 1 2 3 |  |
| Vomiting | 0 1 2 3 | 0 1 2 3 |  |
| Constipation | 0 1 2 3 | 0 1 2 3 |  |
| Diarrhoea | 0 1 2 3 | 0 1 2 3 |  |
| Abdominal pains | 0 1 2 3 | 0 1 2 3 |  |
| Dry mouth | 0 1 2 3 | 0 1 2 3 |  |
| Dizziness | 0 1 2 3 | 0 1 2 3 |  |
| Headaches | 0 1 2 3 | 0 1 2 3 |  |
| Insomnia | 0 1 2 3 | 0 1 2 3 |  |
| Nightmares | 0 1 2 3 | 0 1 2 3 |  |
| Skin rash or itch | 0 1 2 3 | 0 1 2 3 |  |
| Cough | 0 1 2 3 | 0 1 2 3 |  |
| Ankle swelling | 0 1 2 3 | 0 1 2 3 |  |
| Dry eyes | 0 1 2 3 | 0 1 2 3 |  |
| Anaemia | 0 1 2 3 | 0 1 2 3 |  |
| Jaundice | 0 1 2 3 | 0 1 2 3 |  |
| Lipodystrophy | 0 1 2 3 | 0 1 2 3 |  |
| Other: | 0 1 2 3 | 0 1 2 3 |  |
| **TOTAL** |  |  |  |
